# Effectiveness of treatment of newly diagnosed hypertension in family medicine practices in South Croatia

**DOI:** 10.1186/s12875-019-0902-2

**Published:** 2019-01-14

**Authors:** Diana Jurić, Ivančica Pavličević, Ana Marušić, Mario Malički, Ivan Buljan, Velen Šarotić, Nataša Mrduljaš-Đujić, Ante Komparak, Miona Vujević, Danijela De Micheli-Vitturi, Pjera Šušnjar, Tina Puljiz, Minka Jerčić, Dario Leskur, Matko Marušić

**Affiliations:** 1 Department of Pharmacology, University of Split School of Medicine, Šoltanska 2, Split, Croatia; 2 Department of Family Medicine, University of Split School of Medicine, Split, Croatia; 3 Family medicine practice, Split, Croatia; 4 Department of Research in Biomedicine and Health, University of Split School of Medicine, Split, Croatia; 5 Family medicine practice, Lećevica, Croatia; 6 Family medicine practice, Postira, Croatia; 7 Family medicine practice, Korčula, Croatia; 8 Family medicine practice, Imotski, Croatia; 9 Family medicine practice, Pučišća, Croatia; 10 Family medicine practice, Runovići, Croatia; 11 Family medicine practice, Donji Muć, Croatia; 12 Department of Pharmacy, University of Split School of Medicine, Split, Croatia

**Keywords:** Blood pressure, Arterial hypertension, Control, Normotension, Urban, Rural, Island, Hypertension treatment

## Abstract

**Background:**

Uncontrolled blood pressure remains an urgent issue in clinical practice worldwide. This study aimed to compare the characteristics and effectiveness of hypertension control in family medicine pratice in the first treatment year, in relation to the geographical position, socio-economic standard, and access to medical services and public pharmacies in urban, rural and island environments (city of Split vs. Dalmatian Hinterland vs. islands in Southern Croatia).

**Methods:**

A historical cohort study included 213 patients diagnosed from 2008 to 2014 with essential arterial hypertension (AH) and without related complications or diabetes mellitus. Each patient was followed up for 365 days from the visit when the diagnosis of hypertension was ascertained. Normotension was defined as arterial pressure < 140/90 mmHg. The annual cost of drugs prescribed for treating newly diagnosed hypertensive patient and the total price for defined daily dose per patient were also evaluated.

**Results:**

More than half patients achieved normotension within a year from the initial diagnosis in all family medicine practices (57.3%), without significant differences among the three geographic regions (*P* = 0.981). Higher initial systolic blood pressure was a positive predictive prognostic factor on achieveing normotension (odds ratio (OR) 0.96, 95% confidence interval 0.95–0.98). ACE inhibitors were the most commonly prescribed antihypertensive agents in monotherapy (35.1%), as well as considering overall prescriptions (25.2%). Calcium channel blockers were the most commonly prescribed initial BP-lowering single agents in urban areas (28.6%), whereas angiotensin-converting enzyme inhibitors were more common in rural (28.0%) and island areas (22.7%) (*P* = 0.037). The median annual antihypertensive drug cost was 169.4 (95% CI 151.5–201.8) Croatian kunas and was similar across the study sites.

**Conclusion:**

Multiple antihypertensive drugs, prescribed in accordance with the guidelines, lead to similar pharmacological effects. Primary care physicians seem to be able to overcome potential interfering socio-economic factors and successfully achieve normotension in newly diagnosed patients with uncomplicated AH after 1 year of treatment.

**Electronic supplementary material:**

The online version of this article (10.1186/s12875-019-0902-2) contains supplementary material, which is available to authorized users.

## Background

High systolic blood pressure (BP) was one of the most frequent Level 3 risk factors for global burden of disease according to the systematic analysis published in 2016 [1]. The estimated number of adults with elevated BP in 2015 increased to 1.13 bilion, in comparison to 594 milion in 1975 [2]. A 2015 systematic review and meta-analysis, which included data on almost 1.5 million adults from 45 countries, reported 32.3% overall prevalence of arterial hypertension (AH) [3]. Similar prevalence of 30–55% was also found in other recent studies [4–10]. The factors influencing the variations in AH prevalence may be related to the geographical region, degree of urbanization and changing lifestyles [11]. The prevalence in rural areas fluctuate from very low in some studies [12, 13] to significantly higher than in urban areas in the others [14, 15]. Strict BP control in order to prevent cardiovascular morbidity and mortality is recommended in all guidelines for the management of AH [16–20]. Most of data on BP control are obtained from population-based studies, national surveys or secondary care [21], whilst the data on assessment of regulating AH in the primary care settings are sparse [22–24]. However, the data collected in the 2010 National Ambulatory Medical Care Survey, in which hypertension was identified as the most frequently diagnosed condition at physician practice visits, underline the importance of primary health care in prevention, detection and control of high blood pressure [25]. In 2001, cardiovascular diseases were the most common chronic diseases recorded among Croatian family medicine practices [26]. Croatia is among top-ranked countries worldwide according to the prevalence of hypertension, with the highest mean systolic BP in men in 2015 reaching 137.5 mmHg (95% CI 131.2–143.8) [2]. The aim of our study was to assess the treatment and control of uncomplicated AH at the primary health care level in the context of different impacting conditions, such as better transportation accessibility, higher socio-economic standard and educational level in urban settings vs. geographically isolation and poorer access to medical services and public pharmacies in hinterland rural areas and islands of South Croatia.

## Methods

### Study participants and data collection

This historical cohort study included patients of either sex, aged ≥18, whose arterial hypertension was first diagnosed from 2008 to 2014. According to clinical practice guidelines for the managament of AH valid in the time of our research, participants with the mean systolic arterial pressure ≥ 140 mmHg and/or diastolic arterial pressure ≥ 90 mmHg were classified as hypertensive [16–19], and included in the study. Sitting blood pressure had to be estimated in family medicine practices (except in cases of medical specialists’ involvement in the diagnosis) based on a standardized protocol using oscillometric or auscultatory semiautomatic sphygmomanometers [17].

Hypertensive patients were not included in the study if they had 1) AH diagnosed in the period before 2008, 2) incomplete medical records for the studied period, and 3) serious acute and chronic diseases (acute myocardial infarction, cerebrovascular insult and malignant tumors, respectively). Since various abnormalities present in diabetes mellitus and arterial hypertension have significant pathogenetic contribution to the development and exacerbation of each other [27, 28], and because diabetes mellitus is more than twice prevalent in patients with essential hypertension than in normotensive patients [29, 30], patients with diabetes mellitus present prior to the diagnosis of arterial hypertension were excluded from the study.

The data were collected from family medicine offices files. Included practices were long-standing and led by a single-physician, who had individual contracts with the mandatory national Croatian Health Insurance Fund (CHIF). Family medicine practices in Croatia provide health care for an average 1840 patients, with minimum 1275 and maximum 2125 patients allowed [31].

Each patient was followed for 1 year after the initial diagnosis. The urban area included the city of Split and the rural area included the town of Imotski and municipalities Lećevica, Muć, and Runovići (Dalmatian Hinterland). Southern Croatian islands of Brač (municipalities Postira and Pučišća) and Korčula (town of Korčula) represented the island environment (Table 1). The main demographic data included the place of residence (urban, rural, island), age, gender, employment status, marital status, educational level and comorbidities.

**Table 1:** List of family medicine practices included in the study^a^

Family medicine practice	Total number of included patients	Total number of insured^b^	Total population^b^
Urban 1: Split 1	18	1509	178,102
Urban 2: Split 2	46	1851	
Hinterland 1: Imotski	17	2267	10,764
Hinderland 2: Lećevica	38	326	583
Hinterland 3: Donji Muć	8	859	3882
Hinterland 4: Runovići	15	1781	2416
Island 1: Postira	31	1259	1559
Island 2: Pučišća	14	908	2171
Island 3: Korčula	26	1026	5663

^a^Source: Portal of the Open Data of the Republic of Croatia and the Census of Population, 2011, Croatian Central Bureau of Statistics

^b^Data from the Croatian Census of Population, 2011

### Main outcome measures

The primary outcome measures were: 1) rate of achieving normotension after one-year treatment, 2) mean arterial pressure (MAP) before and after one-year treatment, and 3) complications of treatment, including cardiovascular complications and side effects of medications. The achieved blood pressure control was defined as the proportion of participants whose average systolic and diastolic blood pressure 365 days after the initial diagnosis (with a deviation of ±15 days, depending on the scheduled patient visits to the family medicine office) was less than 140/90 mmHg. The secondary outcome measures were: 1) choice of initial treatment, 2) diagnosis by a physician or a medical specialist (usually an internist), 3) length of sick leave days, 4) full or partial coverage of prescribed medications by the national insurance fund, CHIF, 5) total price for defined daily dose (DDD) of all prescribed medications per each patient, and 6) total annual cost of prescribed drugs for treating one newly diagnosed hypertensive patient.

CHIF provides the mandatory health insurance in Croatia, with the obligatory health insurance contributions of employed citizens as the major source of its revenue [32]. This public fund is responsible for health policy implementation, funding and control of health services [33]. There are two reimbursement lists compiled by the CHIF, the Croatian National Medicine Basic Reimbursement List, with both hospital and retail prescription drugs, fully covered by mandatory insurance, and the Croatian National Medicine Supplementary Reimbursement List, where patients have to pay the fraction of the cost, from 10 to 90%. Reimbursement decisions are made according to the criteria established in the Ordinance of Ministry of Health from 2013 [34]. Drugs covered by mandatory insurance can be prescribed only by primary care physicians in the public sector who are contractual partners with the CHIF [26].

To estimate the cost of antihypertensive drugs prescribed during 1 year, original drug packacking prices (including VAT) and prices for defined daily dose (DDD) were taken from the Basic and the Supplementary List, which were valid during data extraction, both in force since 30 May 2015 until 19 July 2015 for the Basic List and until 19 August 2015 for the Supplementary List [35]. The total annual cost of prescribed medications was calculated from the original drug packaging price (including VAT) and the number of prescribed original drug packagings, and the total price for DDD was defined as the sum of all prices for DDD of all medications prescribed to each patient during one-year pharmacotherapy.

We also recorded whether the initial antihypertensive treatment focused on lifestyle changes (Dietary Approaches to Stop Hypertension) [36] without concomitant drug therapy, or whether pharmacological treatment was started immediately after the diagnosis. Prescribed first-line BP-lowering agents were compared with the recommendations from clinical practice guidelines [16–20]. The data on the total number of daily pills (both antihypertensives and non-anithypertensive drugs for all other present comorbidities) and the annual number of drugs per patient were collected from patients’ medical records.

### Statistical analysis

The data were presented as a median or a mean, with 95% confidence interval (CI), depending on the data distribution; the normality of distribution was checked using the Kolmogorov-Smirnov tests. For the comparison of categorical variables we used χ^2^ test. Patients’ age, MAP before and after one-year treatment, number of visits to the family medicine office and sick leave days were compared according to the patients’ place of residence, using analysis of variance (ANOVA) with the post-hoc Scheffé test. Kruskal-Wallis test and post-hoc Mann-Whitney U test were used for the median comparison of the total annual number of prescribed medications per patient and the total annual cost of drugs prescribed for treating one newly diagnosed hypertensive patient by a physician. Mann-Whitney and χ^2^ test were used to determine which variables were significantly different in controlled and uncontrolled hypertensive patients; those variables were further analysed by logistic regression. *P*-values less than 0.05 were considered statistically significant. All statistical analyses were performed using MedCalc, version 12.5 (MedCalc Software, Mariakerke, Belgium).

## Results

### Characteristics of newly diagnosed hypertensive patients

The final study sample included 213 patients; 64 (30.0%) from urban, 78 (36.6%) from rural and 71 (33.3%) from island environment of South Croatia (Table 1). Female patients were predominant in the urban area (*n* = 41, 64.1%), where patients had significantly higher prevalence of dyslipidemia, the most common comorbidity (50.0% vs. 12.1% in the rural area and 26.1% on the islands; *P* = 0.003, Table 2). The rural area had almost four times more unemployed patients than in other areas (*P* < 0.001) and the highest rate of patients with only primary education (*P* = 0.004, Table 2).

**Table 2:** General features of hypertensive patients in urban, rural and island environment of South Croatia

General features	Urban area (*n* = 64)	Rural area (*n* = 78)	Island area (*n* = 71)	*P* ^a^
Age; years (mean, 95% CI)	60 (57–63)	57 (54–59)	57 (54–60)	0.319^b^
No data (n, (%))	0 (0.0)	1 (1.3)	1 (1.4)	
Sex (n, (%)				
Men	23 (35.9)	45 (57.7)	39 (54.9)	0.022
Women	41 (64.1)	33 (42.3)	32 (45.1)	
Employment status (n, (%))				
Employed	24 (37.5)	23 (29.5)	40 (56.3)	< 0.001
Unemployed	6 (9.4)	34 (43.6)	6 (8.5)	
Pensioner	31 (48.4)	21 (26.9)	24 (33.8)	
No data	3 (4.7)	0 (0.0)	1 (1.4)	
Marital status (n, (%))				
Married	39 (60.9)	51 (65.4)	52 (73.2)	0.283
Divorced	3 (4.7)	1 (1.3)	0 (0.0)	
Unmarried	7 (10.9)	15 (19.2)	11 (15.5)	
Widow/er	11 (17.2)	10 (12.8)	8 (11.3)	
No data	4 (6.3)	1 (1.3)	0 (0.0)	
Educational level (n, (%))				
Primary education	7 (10.9)	29 (37.2)	18 (25.4)	0.004
High school graduate	39 (60.9)	43 (55.1)	38 (53.5)	
Postsecondary diploma	3 (4.7)	2 (2.6)	10 (14.1)	
University degree	7 (10.9)	4 (5.1)	4 (5.6)	
No data	8 (12.5)	0 (0.0)	1 (1.4)	
Comorbidities (n, (%))				
Multiple comorbidities	17 (50.0)	29 (87.9)	17 (73.9)	0.003
Dyslipidemia	17 (50.0)	4 (12.1)	6 (26.1)	

^a^χ^2^ test

^b^ANOVA

### Hypertension control

The patients in the whole sample showed a significant reduction in systolic and diastolic blood pressure (paired samples T-test, P < 0.001). There were no differences in the participants with average systolic and diastolic blood pressure lower than 140/90 mmHg: 37 (57.8%) in urban, 45 (57.7%) in rural and 40 (56.3%) in island environment (*P* = 0.981, Table 3). Only the diastolic blood pressure differed after 1 year and was the lowest in island areas (82 mmHg (80.8–84.0 mmHg) vs. 85 mmHg (83.1–86.8 mmHg) in rural areas and 86 mmHg (84.4–87.6 mmHg) in the city; *P* = 0.012, Table 3). Medical specialists from secondary or tertiary health care settings more often partcipated in the diagnosis and/or prescribing the first medication in the urban area (*n* = 13, 20.3%) compared to the rural (*n* = 4, 5.1%) and island areas (*n* = 8, 11.3%) (*P* = 0.020, Table 4). There were no differences regarding general features of patients who achieved normotension and who did not (see Additional file 1: Table S1), although significantly higher proportion of patients had normal BP after 1 year (*P* = 0.034). Analysis of the outcome variables in logistic regression model indicated higher initial systolic blood pressure as a positive predictive prognostic factor on achieveing normotension (OR 0.96, 95% CI 0.95–0.98, R^2^ = 0.06323).

**Table 3:** Primary outcome measures for assessment of the effectiveness of AH treatment in South Croatia

Primary outcome measure	Urban area (n = 64)	Rural area (n = 78)	Island area (n = 71)	*P*
Rate of acheiving normotension (n, (%))	37 (57.8)	45 (57.7)	40 (56.3)	0.981^a^
Arterial pressure [mmHg; mean, 95% CI] before treatment:				
Systolic arterial pressure	158.9 (156.1–161.7)	162.4 (158.8–165.9)	159.2 (155.2–163.2)	> 0.05^b^
Diastolic arterial pressure	96.0 (94.0–97.9)	97.5 (95.5–99.5)	97.8 (95.7–99.8)	> 0.05^b^
Arterial pressure [mmHg; mean, 95% CI] after one-year treatment^c^:				
Systolic arterial pressure	141.0 (138.1–143.8)	137.5 (134.7–140.2)	136.6 (134.1–139.2)	0.073^b^
Diastolic arterial pressure	86.0 (84.4–87.6)	84.9 (83.1–86.8)	82.4 (80.8–84.0)	0.012^b^
Treatment complications (n, (%))	4 (6.3)	7 (9.0)	6 (8.5)	0.838^a^
No data	1 (1.5)	0 (0.0)	0 (0.0)	

^a^χ^2^ test

^b^Two-way ANOVA

^c^Because of the historical study design this time had a deviation of ±15 days

**Table 4:** Secondary outcome measures for assessment of the effectiveness of AH treatment in South Croatia

Secondary outcome measures	Urban area (*n* = 64)	Rural area (*n* = 78)	Island area (*n* = 71)	*P* ^a^
Initial treatment (n, %):				
Non-pharmacological	14 (21.9)	8 (10.3)	18 (25.4)	0.043
Pharmacological	50 (78.1)	70 (89.7)	52 (73.2)	
No data	0 (0.0)	0 (0.0)	1 (1.4)	
Prescribing first medication (n, %):				
Physician	51 (79.7)	74 (94.9)	62 (87.3)	0.020
Medical specialist	13 (20.3)	4 (5.1)	8 (11.3)	
No data	0 (0.0)	0 (0.0)	1 (1.4)	
Sick leave days beacause of arterial hypertension (mean, 95% CI)				
Total number of days^b^	1.8 (0.0–4.2)	3.6 (0.0–10.0)	1.2 (0.0–2.6)	0.692^c^
Proportion (n, %) of patients with prescribed medications from the List(s)^d^				
Basic	35 (64.8)	49 (70.0)	36 (60.0)	0.286
Supplementary	11 (20.4)	6 (8.6)	9 (15.0)	
Basic + Supplementary	8 (14.8)	15 (21.4)	15 (25.0)	
Total price for DDD per patient (median, HRK, interquartile range)^e^				
Total price for DDD	0.91 (0.49–1.24)	0.96 (0.45–1.55)	1.19 (0.49–2.06)	0.228^f^

^a^χ^2^ test

^b^Calculated only for employed patients

^c^ANOVA

^d^Excluded participants without pharmacological treatment during the first treatment year (n = 16) and with incomplete data on medications required for analysis (n = 13)

^e^The median of all calculated sums of prices for defined daily dose (DDD) in Croatian kuna (HRK) of all medications prescribed to each patient during one-year treatment. Excluded participants without pharmacological treatment during the first treatment year (*n* = 16) and with incomplete data on medications required for analysis (*n* = 13)

^f^Kruskal-Wallis test

### Cost of prescribed drugs

The median annual cost of antihypertensive drugs for treating newly diagnosed hypertensive patient was 169.4 (95% CI 151.5–201.8) Croatian kunas. Only a single physician from the rural area had significantly higher annual cost than the others (290.3, 95% CI = 214.1–394.7 Croatian kunas, *P* < 0.001). Individual physicians did not differ in prescribing medications from two Croatian National Medicine Reimbursement Lists (*P* = 0.286, Table 4) and in the total cost for defined daily dose of all prescribed medications per each patient (*P* = 0.228, Table 4).

### Drug prescriptions

Pharmacotherapy approaches to the first-line treatment differed in urban, rural and island environment (*P* = 0.037, Table 5). Calcium channel blockers were the most commonly prescribed initial BP-lowering single agents in urban areas (*n* = 16, 28.6%), whereas angiotensin-converting enzyme inhibitors (ACEI) were more common in rural (*n* = 21, 28.0%) and island areas (*n* = 15, 22.7%).

**Table 5:** Characteristics of antihypertensive pharmacotherapy in urban, rural and island environment of South Croatia

Pharmacotherapy	Urban area (*n* = 56)^a^	Rural area (*n* = 75)^a^	Island area (*n* = 66)^a^	*P* ^b^
First-line treatment (n, (%))				
1. Diuretics	3 (5.4)	8 (10.7)	1 (1.5)	0.037
2. ACE inhibitors (ACEI)	14 (25.0)	21 (28.0)	15 (22.7)	
3. Calcium channel blockers (CCB)	16 (28.6)	5 (6.7)	9 (13.6)	
4. Angiotensin II receptor blockers (ARB)	0 (0.0)	0 (0.0)	1 (1.5)	
5. β-adrenergic receptor blockers (BB)	5 (8.9)	8 (10.7)	14 (21.2)	
7. Fixed-dose combination:^c^	8 (14.3)	12 (16.0)	7 (10.6)	
ACEI + diuretic	5 (8.9)	9 (12.0)	5 (7.6)	
BB + diuretic	2 (3.6)	0 (0.0)	2 (3.0)	
ARB + diuretic	0 (0)	1 (1.3)	0 (0.0)	
ACEI + CCB	1 (1.8)	2 (2.7)	0 (0.0)	
8. > 1 medications (2 or 3)^c^	10 (17.9)	21 (28.0)	19 (28.8)	
One-year treatment (median, 95% CI)				
Total daily number of pills per patient^d^	1.5 (1.0–2.0)	1.5 (1.0–2.0)	2 (1.0–2.6)	0.217
Total annual number of drugs per patient	1 (1.0–2.0)	2 (1.0–2.0)	1 (1.0–2.0)	0.135^e^

^a^Excluded participants without pharmacological treatment during the first treament year (*n* = 16)

^b^χ^2^ test

^c^Fixed-dose combination of two active pharmaceutical ingredients is considered as one drug

^d^Calculated average total daily number of pills per each patient (since the number of pills varied during a following year) was used to calculate the median of total daily number of pills for urban, rural and island enironment

^e^Kruskal-Wallis test

Within the first year of therapy, a total of 77 patients (39.1%) out of 197 patients who received pharmacological therapy received monotherapy, whereas 120 patients (60.9%, Table 6) were on multiple drug therapy. In the monotherapy group, ACEI were the most commonly prescribed (*n* = 27, 35.1%), β-blockers ranked second (*n* = 22, 28.6%), followed by calcium channel blockers (*n* = 18, 23.4%) and thiazide-like diuretics (*n* = 9, 11.7%, Table 6). Among 54 patients who achieved normotension with monotherapy, lisinopril, amlodipine and bisoprolol were the most frequently prescribed (*n* = 11, 20.4% for each, respectively).

**Table 6:** Prescription rates in treating newly diagnosed arterial hypertension in South Croatia

Prescribed antihypertensive class	Proportion of medication prescribed, n, (%)	
	Monotherapy^a^	Overall prescriptions^a^
Diuretics:	9 (11.7)	55 (16.7)
Thiazide-like diuretics	9 (11.7)	48 (14.6)
Loop diuretics	0 (0.0)	6 (1.8)
Aldosterone antagonists	0 (0.0)	1 (0.3)
Angiotensin-converting enyzme inhibitors^a^	27 (35.1)	83 (25.2)
Calcium channel blockers	18 (23.4)	57 (17.3)
Angiotensin II receptor blockers	1 (1.3)	11 (3.3)
Antiadrenergic agents^b^	22 (28.6)	61 (18.5)
Fixed-dose combinations	/	62 (18.8)

^a^Excluded: patients without any pharmacological treatment (n = 16). There were 77 patients receiving only one antihypertensive drug during the first treament year. A total of 330 drugs were prescribed to 197 patients during one year and considered for the overall utilization, including mono and combined therapies, that were at least once prescribed to one newly diagnosed hypertensive during one-year treatment. The required data for one ACE inhibitor was missing, leaving 329 drugs for analysis

^b^Only beta-adrenergic blocking agents in the monotherapy group (*n* = 22). Beta blockers were predominant overall (*n* = 60), only one was selective alpha-2 adrenergic and imidazoline receptor agonist

In the case of overall pattern of all prescribed antihypertensive agents (mono and combined therapies, 329 drugs in total), ACEI were again the most commonly prescribed (*n* = 83, 25.2%), followed by β-adrenergic receptor antagonists (*n* = 60, 18.2%), calcium channel blockers (*n* = 57, 17.3%) and diuretics (*n* = 55, 16.7%, Table 6). Among the ACEI, lisinopril (*n* = 41, 49.4%) and ramipril (*n* = 25, 30.1%) were the most widely prescribed. Thiazide-like diuretics, chlortalidone (*n* = 30, 54.5%) and indapamide (*n* = 18, 32.7%), were the most commonly used among single-agent diuretics. Thiazide diuretics were prescribed only in fixed-dose combinations that were available on the Croatian drug market. Among fixed-dose combinations, the most frequently applied in the first year of treatment was lisinopril in combination with hydrochlorothiazide (*n* = 23, 37.1%).

Prescribed anthypertensive drug classes did not differ among patients who achieved control of AH and those who did not (*P* = 0.205, see Additional file 1: Table S2). Lisinopril (*n* = 27, 15.4%) and bisoprolol (*n* = 22, 12.6%, see Additional file 1: Table S3) were the most commonly used considering 115 patients which were normotensive after 1 year of pharmacological treatment.

## Discussion

Our study demostrated that the sucess in controlling uncomplicated essential hypertension in a family medicine setting within the first year of treatement was relatively high (57.3%) and similar in different geographical settings: in the second largest city in Croatia (Split) and its more geographically isolated hinterland and islands, regardless of socioeconomic inequalities [37, 38], access to health care and differences in prescribing patterns. Our findings are in contrast with reported positive association between socioeconomic variety and normotension rates found in most international studies [14, 39–41], as well as frequent insufficient hypertension managament in general practice [42, 43].

In an indirect manner, these findings correspond to a recent study in this region, which found that general health status in this area did not differ with respect to the geographical and socioeconomic factors [37]. It is important to emphasize that our study addressed only patients with uncomplicated hypertension treatment. We excluded the patients with diabetes since it was shown to be associated with higher systolic and diastolic BP values [44] and a negative predictor of BP control [45]. The rates of achieving normotension were relatively high, compared to 32.5% of the patients receiving treatment and controlled in the international community-based Prospective Urban Rural Epidemiology (PURE) study [39]. However, our results are generally consistent with the findings from recent European studies [46–48] and demonstrate an increase in the rate of achieving the targeted BP in Croatia when compared to the rate of 19.4% from the EH-UH study published in 2007 [49]. However, it has to be highlighted that in almost all of the aforementioned studies, study population included diabetic hypertensive patients [39, 41, 44–49]. In a comparative study of three different European communities, England, Belgium and Italy, with diabetes mellitus as one of exclusion criteria, the rates of achieving normotension among those treated were 42.0, 43.0 and 47.7%, respectively [50]. Canadian middle-aged hypertensive patients without diabetes mellitus or cardiovascular diseases had almost similar sucess rate of 57.4% [51].

The second important finding of our study was that the different antihypertensive drugs and treatment choices led to similar effectiveness in BP control. ACE inhibitors were the most widely prescribed antihypertensive single drug therapy in the first-line in rural (28.0%) and island areas (22.7%), whilst calcium channel blockers predominated in urban setting (28.6%). The choice of initial treatment was in line with the 2013 guidelines of the European Society of Hypertension (ESH) and European Society of Cardiology (ESC), which recommended the following five pharmacotherapeutic groups for the initiation and maintenance of antihypertensive treatment – diuretics, β-blockers, calcium channel blockers, ACE inhibitors and angiotensin receptor II blockers, as monotherapy or combined polytherapy [17]. However, there was a significant disagreement with the recommendations of most other international guidelines, valid in the time of conducting our research, for the β-blockers, which were, except in 2013 ESH/ESC guidelines [17], mainly recommended as a third [19] or a fourth-line treatment [16, 18, 20]. The first-line β-blockers were not prominent in the Eighth Joint National Committee (JNC 8) guidelines from 2014 [20], due to higher rate of the primary composite outcome of cardiovascular death, myocardial infarction, and increased risk of stroke compared to use of losartan [52] and other antihypertensive drugs [53]. The proportion of β-blockers in the initial treatment was higher on the islands (21.2%), in comparison with Dalmatian Hinterland (10.7%) and Split (8.9%). It is interesting that physicians in urban and rural environment replaced beta-blockers with recommended first-line therapy options, whilst island family physicians sustain this older therapeutic approach [54]. Possible reasons underlying this practice are not clear and should be further investigated.

Diuretics are the first-line therapy choice for most hypertensive patients according to the recommendations of the Seventh Joint National Committee (JNC 7) guidelines [55] as well as guidelines of the World Health Organization and the International Society of Hypertension from 2003 [56]. Thiazide-like diuretics [16, 17, 20] and thiazide diuretics [17–19] are initially recommended in almost all mentioned international guidelines for the management of arterial hypertension. In spite of that, diuretics administered alone in the first-line were prescribed to only 1.5% of the patients at practices on the Croatian islands, 5.4% in the city of Split and 10.7% in the rural area (Table 5). It has been shown that most general practitioners consider diuretics as too weak to treat arterial hypertension effectively in monotherapy [57], which could support this prescribing pattern in South Croatia, and they appear to be initial antihypertensive class with the lowest persistance rate as well [58]. Nonetheless, it should be mentioned that the availability of thiazide diuretics only in fixed-dose combinations on the drug market in Croatia could bias our results, limiting physicians’ choice to two-drug regimen immediately after the initial diagnosis. The higher proportion of diuretics in the initial treatment in the rural setting is in line with the results from the study conducted in Split and Trilj, another representative of Dalmatian rural area (2005–2010) [59]. The ingrained perception of diuretics as inexpensive drugs [60] could be one of possible reason underlying this primary care prescribing behavior in the rural area with assumed lower socioeconomic status.

Since better BP reduction is often achieved by combining drugs from two different classes in comparison with doubling the dose of one drug [61] and with fixed-dose combinations there is also significant improvement in the compliance [62], the frequency of fixed-dose combinations could have been higher (18.8% during the first year of treatment). Hence, it might be required to further investigate reasons of relatively low rates of prescribing fixed-dose combinations among physicians in order to increase the rate of achieving normotension even more.

Taking into account that we found only a single physician from rural area with significantly higher total annual cost of prescribed drugs for treating one newly diagnosed hypertensive patient, it is difficult to make judgements on differences in the costs of AH treatment across different regions. However, comparing the highest annual average antihypertensive drugs cost in this study (290.3 Croatian kunas, approximately 38.9€, 95% CI = 214.1–394.7 Croatian kunas) with the cost of €258 during follow-up period of 36 ± 3 months in French general practices [63], it could be concluded that the cost of drugs for treating high blood pressure in Croatia is relatively low. Our findings suggest that different BP-lowering agents have similar financial effects, but the small sample sizes did not allow stronger conclusions. Despite noticed decreasing trend of the primary care expenditure considering the overall healthcare expenditure in Croatia from 1989 to 2000 [26], there were no differences among physicians in prescribing drugs from two Croatian National Medicine Reimbursement Lists.

Primary care is an important interface between the patients and the health care system, shown to be associated with improved health outcomes [64]. The results of our study indicate that family medicine physicians in Croatia could be the cornerstone in controlling arterial hypertension. However, discordance found comparing international hypertension prevalence rates and recorded prevalence in family medicine practices in Croatia, which was lower than 10% in 2001 [26], emphasize the need for more effective coverage of the population by family medicine services, and the improvement of screening programmes and detection at the primary care level.

The key strenght of this study lies in its comprehensive analysis of the effectiveness of newly diagnosed arterial hypertension treatment in primary care setting. It provides insight into the impact of primary care in the achieving control of uncomplicated essential hypertension in the real-life socio-economic conditions. The major limitation of the study is its retrospective character. Furthermore, due to the inability to access two Croatian National Medicine Reimbursement Lists valid in time periods that involved one-year following intervals, original drug packaging prices were taken from the Croatian National Medicine Reimbursement Lists which were valid during the data extraction, in force since May 2015 [35]. Finally, the calculation of direct cost of hypertension treatment should include all other medical, as well as non-medical direct costs [65] to assess the full the cost of treating AH.

## Conclusion

To conclude, there is a remarkable lack of association between socioeconomic conditions and uncomplicated arterial hypertension control in primary care setting. Different antihypertensive prescribing patterns seem do not have an impact on the effectiveness in blood pressure control after the one-year treatment. Family physicians seem to be able, without any intervention by medical specialists, to achieve normotension in newly diagnosed patients with arterial hypertension, emphasizing their key role in dealing with this major public health problem.

## Additional file

Additional file 1: **Table S1.** General characteristics of patients according to their blood pressure control after one-year treatment. **Table S2.** Prescribed pharmacological subgroups for patients with and without achieved blood pressure control after one-year treatment. **Table S3.** Prescribed generic names for patients with and without achieved blood pressure control after one-year treatment. (DOCX 68 kb)

## Abbreviations

ACEI: Angiotensin-converting enzyme inhibitor
AH: Arterial hypertension
ANOVA: Analysis of variance
ARB: Angiotensin receptor II blockers
BB: β-adrenergic receptor antagonists
BP: Blood pressure
CCB: Calcium channel blockers
CHIF: Croatian Health Insurance Fund
CI: Confidence interval
DDD: Defined daily dose
EH-UH Study: Epidemiology of Hypertension in Croatia
ESH/ESC: European Society of Hypertension/European Society of Cardiology
HRK: Croatian kuna
JNC 7: Seventh Joint National Committee
JNC 8: Eighth Joint National Committee
MAP: Mean arterial pressure
OR: Odds ratio
PURE: Prospective Urban Rural Epidemiology study
VAT: Value-added tax
