# Supplementary material for: Effectiveness of treatment of newly diagnosed hypertension in family medicine practices in South Croatia
Source: BMC Fam Pract. 2019 Jan 14;20:10. doi: 10.1186/s12875-019-0902-2 (PMC6330736; doi:10.1186/s12875-019-0902-2)
Supplement: Supplementary file 1 — Table S1. General characteristics of patients according to their blood pressure control after one-year treatment. Table S2. Prescribed pharmacological subgroups for patients with and without achieved blood pressure control after one-year treatment. Table S3. Prescribed generic names for patients with and without achieved blood pressure control after one-year treatment. (DOCX 68 kb) [file 12875_2019_902_MOESM1_ESM.docx]

**Additional file 1**

**Table S1** General characteristics of patients according to their blood pressure control after one-year treatment.

**Table S2** Prescribed pharmacological subgroups for patients with and without achieved blood pressure control after one-year treatment.

**Table S3** Prescribed generic names for patients with and without achieved blood pressure control after one-year treatment.

**Table S1** General characteristics of patients according to their blood pressure control after one-year treatment.

| General features | Achieved normotension* | Sustained hypertension * | P† |
| --- | --- | --- | --- |
| Living place (n, (%)) |  | |  |
| Urban area | 37 (30.3) | 27 (29.7) | 0.981 |
| Rural area | 45 (36.9) | 33 (36.3) |  |
| Island area | 40 (32.8) | 31 (34.1) |  |
| Age |  | |  |
| Years (median, 95 % CI) | 57 (55 – 59) | 55 (54 – 60) | 0.829‡ |
| No data (n, (%)) | 2 (100.0) | 0 (0.0) |  |
| Sex (n, (%)) |  | |  |
| Men | 58 (47.5) | 49 (53.8) | 0.364 |
| Women | 64 (52.5) | 42 (46.2) |  |
| Employment status (n, %) |  | |  |
| Employed | 52 (42.6) | 35 (38.5) | 0.290 |
| Unemployed | 29 (23.8) | 17 (18.7) |  |
| Pensioner | 38 (31.1) | 38 (41.8) |  |
| No data | 3 (2.5) | 1 (1.1) |  |
| Marital status (n, %) |  | |  |
| Married | 83 (68.0) | 59 (64.8) | 0.055 |
| Divorced | 4 (3.3) | 0 (0.0) |  |
| Unmarried | 13 (10.7) | 20 (22.0) |  |
| Widow/er | 18 (14.8) | 11 (12.1) |  |
| No data | 4 (3.3) | 1 (1.1) |  |
| Educational level (n, %) |  | |  |
| Primary education | 34 (27.9) | 20 (22.0) | 0.605 |
| High school graduate | 65 (53.3) | 55 (60.4) |  |
| Postsecondary diploma | 7 (5.7) | 8 (8.8) |  |
| University degree | 9 (7.4) | 6 (6.6) |  |
| No data | 7 (5.7) | 2 (2.2) |  |

*Among 213 included patients, 122 patients (57.3%) achieved normotension after one year of pharmacological therapy and 91 (42.7%) did not.

†χ2 test.

‡Mann-Whitney test for independent samples.

**Table S2** Prescribed pharmacological subgroups for patients with and without achieved blood pressure control after one-year treatment.

| Prescribed antihypertensive class | Proportion of medication prescribed, n, (%)*,† | |
| --- | --- | --- |
|  | Achieved normotension† | Sustained hypertension† |
| Diuretics: | 33 (18.9) | 22 (14.3) |
| Thiazide-like diuretics | 28 (16.0) | 20 (13.0) |
| Loop diuretics | 5 (2.9) | 1 (0.6) |
| Aldosterone antagonists | 0 (0.0) | 1 (0.6) |
| Angiotensin-converting enzyme inhibitors | 46 (26.3) | 37 (24.0) |
| Calcium channel blockers | 23 (13.1) | 34 (22.1) |
| Angiotensin II receptor blockers | 6 (3.4) | 5 (3.2) |
| Antiadrenergic agents | 37 (21.1) | 24 (15.6) |
| Fixed-dose combinations | 30 (17.1) | 32 (20.8) |

*Prescribed drug classes did not differ among patients who achieved blood pressure control and those who did not (P=0.205, χ2 test).

†A total of 330 drugs prescribed to 197 patients during one year were considered for the overall utilization; patients without any pharmacological treatment were excluded (n=16). The required data for one ACE inhibitor was missing, leaving 329 drugs for analysis. One or more antihypertensive drugs were prescribed to 115 patients that were normotensive after one year (in total 175 drugs) and to 82 patients that remained with high blood pressure (in total 154 drugs). Proportions of medication prescribed were calculated for these total number of prescribed drugs.

**Table S3** Prescribed generic names for patients with and without achieved blood pressure control after one-year treatment.

| Prescribed antihypertensive drug | Proportion of medication prescribed, n, (%)*,† | |
| --- | --- | --- |
|  | Achieved normotension† | Sustained hypertension† |
| Diuretics: |  | |
| Indapamide | 11 (6.3) | 7 (4.5) |
| Chlortalidone | 17 (9.7) | 13 (8.4) |
| Torasemide | 1 (0.6) | 0 (0.0) |
| Furosemide | 4 (2.3) | 1 (0.6) |
| Spironolactone | 0 (0.0) | 1 (0.6) |
| ACE inhibitors: |  | |
| Cilazapril | 0 (0.0) | 1 (0.6) |
| Enalapril | 2 (1.1) | 0 (0.0) |
| Lisinopril | 27 (15.4) | 14 (9.1) |
| Perindopril | 1 (0.6) | 1 (0.6) |
| Ramipril | 9 (5.1) | 16 (10.4) |
| Trandolapril | 7 (4.0) | 5 (3.2) |
| Calcium channel blockers: |  | |
| Amlodipine | 19 (10.9) | 25 (16.2) |
| Felodipine | 0 (0.0) | 1 (0.6) |
| Lacidipine | 3 (1.7) | 6 (3.9) |
| Lercanidipine | 0 (0.0) | 1 (0.6) |
| Verapamil | 1 (0.6) | 1 (0.6) |
| Angiotensin II receptor blockers: |  | |
| Losartan | 5 (2.9) | 3 (1.9) |
| Valsartan | 1 (0.6) | 2 (1.3) |
| Antiadrenergic agents: |  | |
| Atenolol | 8 (4.6) | 4 (2.6) |
| Bisoprolol | 22 (12.6) | 15 (9.7) |
| Carvedilol | 1 (0.6) | 0 (0.0) |
| Nebivolol | 5 (2.9) | 2 (1.3) |
| Propranolol | 1 (0.6) | 2 (1.3) |
| Moxonidine | 0 (0.0) | 1 (0.6) |
| Fixed-dose combinations: |  | |
| Bisoprolol + amlodipine | 1 (0.6) | 0 (0.0) |
| Bisoprolol + hydrochlorothiazide | 6 (3.4) | 2 (1.3) |
| Lisinopril + hydrochlorothiazide | 12 (6.9) | 11 (7.1) |
| Losartan + hydrochlorothiazide | 2 (1.1) | 2 (1.3) |
| Perindopril + amlodipine | 2 (1.1) | 4 (2.6) |
| Perindopril + indapamide | 3 (1.7) | 1 (0.6) |
| Ramipril + amlodipine | 1 (0.6) | 0 (0.0) |
| Ramipril + felodipine | 1 (0.6) | 1 (0.6) |
| Ramipril + hydrochlorothiazide | 1 (0.6) | 9 (5.8) |
| Valsartan + hydrochlorothiazide | 0 (0.0) | 1 (0.6) |
| Verapamil + trandolapril | 1 (0.6) | 1 (0.6) |

*Prescribed drug classes did not differ among patients who achieved blood pressure control and those who did not (P=0.205, χ2 test).

†A total of 330 drugs prescribed to 197 patients during one year were considered for the overall utilization; patients without any pharmacological treatment were excluded (n=16). The required data for one ACE inhibitor was missing, leaving 329 drugs for analysis. One or more antihypertensive drugs were prescribed to 115 patients that were normotensive after one year (in total 175 drugs) and to 82 patients that remained with high blood pressure (in total 154 drugs). Proportions of medication prescribed were calculated for these total number of prescribed drugs.
